# Transcriptome Meta-Analysis Confirms the Hidradenitis Suppurativa Pathogenic Triad: Upregulated Inflammation, Altered Epithelial Organization, and Dysregulated Metabolic Signaling

**DOI:** 10.3390/biom12101371

**Published:** 2022-09-25

**Authors:** Ana Sofia Lima Estevao de Oliveira, Giovanna Bloise, Chiara Moltrasio, Antonio Coelho, Almerinda Agrelli, Ronald Moura, Paola Maura Tricarico, Stéphane Jamain, Angelo Valerio Marzano, Sergio Crovella, Lucas André Cavalcanti Brandão

**Affiliations:** 1 Laboratory of Immunopathology Keizo Asami-LIKA, Federal University of Pernambuco, Recife 50670-901, Brazil; 2 Department of Pathology, Federal University of Pernambuco, Recife 50670-901, Brazil; 3 Hospital Israelita Albert Einstein, São Paulo 05652-000, Brazil; 4 Dermatology Unit, Fondazione IRCCS Ca’ Granda Ospedale Maggiore Policlinico, 20122 Milan, Italy; 5 Department of Medical Surgical and Health Sciences, University of Trieste, 34137 Trieste, Italy; 6 Laboratory of Nanostructured Materials (LMNANO), Center for Strategic Technologies Northeastern (CETENE), Av. Prof. Luís Freire, 1-Cidade Universitária, Recife 50740-545, Brazil; 7 Department of Advanced Diagnostics, Institute for Maternal and Child Health-IRCCS “Burlo Garofolo”, 34137 Trieste, Italy; 8 Translational Neuropsychiatry, Univ. Paris Est Créteil, Inserm, IMRB, 94010 Créteil, France; 9 Department of Pathophysiology and Transplantation, Università degli Studi di Milano, 20122 Milan, Italy; 10 Biological Science Program, Department of Biological and Environmental Sciences, College of Arts and Sciences, University of Qatar, Doha 2713, Qatar

**Keywords:** acne inversa, variant enrichment analysis, OMICs

## Abstract

Hidradenitis suppurativa (HS) is an inflammatory skin condition clinically characterized by recurrent painful deep-seated nodules, abscesses, and sinus tracks in areas bearing apocrine glands, such as axillae, breasts, groins, and buttocks. Despite many recent advances, the pathophysiological landscape of HS still demands further clarification. To elucidate HS pathogenesis, we performed a meta-analysis, set analysis, and a variant calling on selected RNA-Sequencing (RNA-Seq) studies on HS skin. Our findings corroborate the HS triad composed of upregulated inflammation, altered epithelial differentiation, and dysregulated metabolism signaling. Upregulation of specific genes, such as *KRT6*, *KRT16*, serpin-family genes, and *SPRR3* confirms the early involvement of hair follicles and the impairment of barrier function in HS lesioned skin. In addition, our results suggest that adipokines could be regarded as biomarkers of HS and metabolic-related disorders. Finally, the RNA-Seq variant calling identified several mutations in HS patients, suggesting potential new HS-related genes associated with the sporadic form of this disease. Overall, this study provides insights into the molecular pathways involved in HS and identifies potential HS-related biomarkers.

## 1. Introduction

Hidradenitis suppurativa (HS), also known as acne inversa, is a chronic inflammatory skin disease involving the pilosebaceous unit, with a worldwide prevalence varying from 0.03% to 4% [1,2,3,4,5,6,7]. HS lesioned skin is typically associated with apocrine gland-bearing body areas, such as axillae, breasts, groins, and buttocks [1,2]. HS is clinically characterized by painful and recurrent deep-seated inflammatory nodules and abscesses [3] that, with disease progression, may evolve into dermal tunnels with malodorous discharge and disfiguring scarring, severely affecting patients’ quality of life [4,5].

HS pathogenesis is still poorly understood, although hereditary factors have demonstrated the ability to increase risks associated with the development of the skin condition. [6]. It has been proposed that the primum movens of the disease are represented by keratin follicular plugging progressing to follicle rupture, with subsequent upregulation of the immune system response that leads to an overexpression of pro-inflammatory cytokines/chemokines, as well as several other pro-inflammatory mediators [7,8,9]. Several genetic changes have been involved in HS pathogenesis, including mutations in the γ-secretase complex genes, both in familial and syndromic forms of HS [10,11,12]. On the other hand, the γ-secretase gene mutations only occur in roughly 6% of sporadic cases of HS [13]. The γ-secretase complex has the notch signaling pathway as a substrate, which has been shown to alter the keratinocyte differentiation program leading to uncontrolled cell proliferation when downregulated [14]. Moreover, both γ-secretase genes and notch signaling pathway play a crucial role in the development of epidermal cysts and comedones, two phenotypic traits of HS [15]. With several new gene pathogenic variants identified, HS can be considered a multifactorial, polygenic, autoinflammatory condition [12].

In the -omic era, the next-generation sequencing (NGS) approach made major contributions to the study of HS [12,16]. Herein, we performed a meta-analysis of selected RNA sequencing (RNA-Seq) studies, comparing HS lesioned and healthy skin. With this integrated meta-analysis approach, it [17] was possible to combine results from independent studies to increase statistical power and obtain a more precise estimate of differentially expressed genes (DEGs). In addition, we carried out pathway analysis to characterize the DEGs in HS pathogenesis. Lastly, a variant calling was performed allowing the identification of expression quantitative trait loci (eQTL) associated with DEGs. Through these analyses performed we explored HS pathogenesis and created a link between candidate genes and their transcripts, genotypes, and clinical phenotypes.

## 2. Materials and Methods

### 2.1. Selection of Publicly Available Studies

We searched Sequence Read Archive (SRA) [18] and Gene Expression Omnibus (GEO) [19] datasets on 11 July 2022, to find studies involving RNA-Seq of HS tissue samples. The search keywords were: ((Hidradenitis Suppurativa) OR (Acne Inversa)) AND ((RNA-Seq) OR (Transcriptome)) [20]. After removing duplicates, we included studies that met the criteria of: experiments carried out in HS patients’ skin (*Homo sapiens* organism filter); had matching healthy skin controls; and had raw data (fastq files) available for each sample. Biopsies collected from lesioned skin of HS patients were considered cases, whereas biopsies collected from the skin of patients without HS, or any other skin condition, were accepted as controls.

### 2.2. RNA-Seq Data Collection, Processing, and Analysis

**Figure 1:**
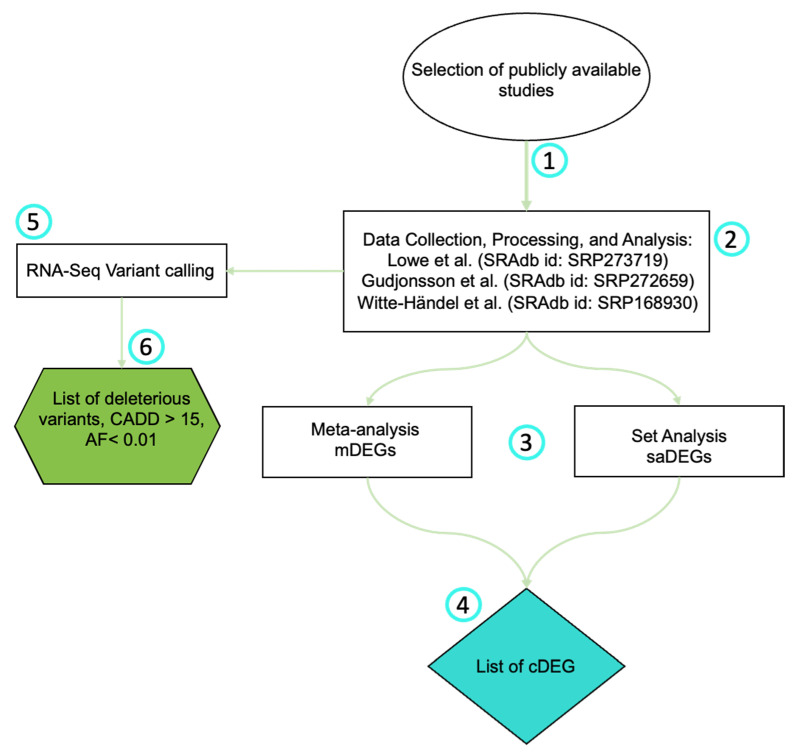
Analysis flowchart: 1: Publicly available studies were selected from the Sequence Read Archive (SRA) and Gene Expression Omnibus (GEO); 2: Three studies passed our selection criteria and were downloaded. SRAdb package for R software version 4.1.0 was used to download all raw. fastq files. Trimmomatic v0.39 was used to trim Illumina adapters and to exclude reads. Resulting gene counts tables were converted into *DESeq2* package objects [37,38,39]; 3: Lists of DEGs found through the meta-analysis (mDEGs) or the set analysis (saDEGs) (See supplementary data) were created; 4: List of common DEGs (cDEGs) found in both analyses was made (See supplementary data); 5: RNA-Seq variant calling analysis was performed by Strelka v2.9.10; 6: A list of potential HS-related variants originated based on deleterious capacity, allele frequency, and significant genotypic variation based on Wilcoxon Test (*p* value < 0.5).

Figure 1 summarizes the methodology. Data collection, processing, and analysis were conducted as previously described by [21,22]. Briefly, SR Adb package [23] for R software version 4.1.0 [24] was used to download all raw. fastq files. Only the sequencing reads that met the study criteria were downloaded. Then, we reprocessed all reads using the same standard workflow to avoid bias due to heterogeneous pipelines of the original studies. The workflow used Trimmomatic v0.39 [25] to trim Illumina adapters, and to exclude low-quality reads (Q < 30) and reads counting shorter than 25 bases (length < 25). Then, the remaining reads were mapped on the National Center for Biotechnology (NCBI) human GRCh38/hg38 reference genome and sorted by coordinates using STAR aligner [26]. Aligned reads (BAM files) were imported into R software and processed with an annotation file from the reference genome with Rsubread package [27]. As a result, a gene counts table was created for each sample. These tables were converted into *DESeq2* package objects, where we screened for DEGs [28]. Genes with log2(fold change) > 1 and false discovery rate (FDR)-adjusted *p*-values < 0.05 were acknowledged as statistically significant DEGs.

### 2.3. Meta-Analysis

We integrated the results produced by the independent groups with a meta-analysis using the RankProd package for R software. The package performs a nonparametric approach based on ranks of fold changes (FC) that detect differentially consistently expressed genes (mDEGs) from independent and replicated experiments. Overall, the meta-analysis approach was based on Lee et al. methodology (2019) [21].

### 2.4. Pathway Analysis

We performed a pathway enrichment analysis with the mDEGs. We searched the REACTOME database, using ReactomePA package [24] for R software, and PANTHER v17.0 [29,30]. As in the previous analyses, results with FDR-adjusted enrichment test *p*-value < 0.05 were considered significant.

### 2.5. RNA-Seq Variant Calling

We performed a variant calling sourcing from the aligned RNA-Seq data. Post-processing was applied using Genome Analysis Toolkit v4.1.2.0 [31] to the aligned reads: duplicates were marked and removed with Mark Duplicates and Split NCigar Reads, and base recalibration was completed with Base Reca librator and Apply BQSR. Variant Calling was performed using Strelka v2.9.10 [32], and single nucleotide polymorphisms (SNPs) and insertions and deletions (INDELs) were separated in two variant calling format (VCF) files using select variants. The resulting variants were removed using Variant Filtration if SNPs had QD < 2.0, FS > 60.0, MQ < 40.0, MQ Rank Sum < −12.5, Read Pos Rank Sum < −8.0 and GQ < 20.0, and if INDELs had QD < 2.0, FS > 200.0, Read Pos Rank Sum < −20.0 and GQ < 20.0. Annotation was performed using Annovar v2019Oct24 [33]. Finally, variants that were present in HS patients were only kept if they had depth (DP > 10) and quality score (QUAL > 30).

To infer the connection between genetic variants and HS pathogenesis, we conducted several approaches. Initially we searched for variants in genes previously associated with HS. Then, we sought variants in new potential HS genes. To unravel the new ones, we selected variants based on some filters detailed as follows. Estimates of HS prevalence vary between 0.03% and 4% among different populations [34,35]. Therefore, it has not been well defined if HS is a rare or a neglected disease [36]. Since rare variants are commonly responsible for disease appearance, herein, for analysis purposes only, we considered HS a rare skin condition (AF ≤ 0.01). Thus, potentially deleterious variants found in genes previously associated with HS or with a CADD (phred) score > 15, possessing clinical significance different from benign or likely benign, based on the Clin Var database, and having statistically significant (*p*-value < 0.05) differences in the genotype were selected as potential HS genetic markers. The median value of the RNA expression level was calculated for each genotype; large differences between the genotypes were considered statistically significant according to the Wilcoxon test.

### 2.6. Statistics

The Shapiro-Wilk test was used to assess if the counts were normally distributed. To uncover if genotypes were associated with differential RNA expression, we used an appropriate test for normally distributed counts based on the number of observed genotypes (normal and two genotypes: Sztudent’s *t*-test for independent samples; not normal and two genotypes: Wilcoxon-Mann-Whitney test; normal and three genotypes: ANOVA; not normal and three genotypes: Kruskal-Wallis test. In the case of three-genotypes tests, we performed a post-hoc paired Wilcoxon test with FDR correction to identify which genotype was responsible for the overall difference).

## 3. Results

### 3.1. Dataset Selection

The search strategy conducted by three authors independently (A.S.L.E.O, G.B., and A.A.) resulted in 25 datasets. Among these, seven studies used microarrays; eight used RNA-Seq; six used quantitative real time polymerase chain reaction (PCR); three used single cell (sc) RNA-Seq; and one used target capture. Since we were searching for RNA-Seq studies only, 17 studies were excluded. Out of the eight RNA-Seq studies, six contained analysis of skin biopsies. Of these six studies, two of them did not have matching healthy skin control, and another analyzed familial HS cases only. Therefore, only three studies were included in our meta-analysis and set analysis. Herein, a total of 51 unique HS lesioned samples, and 30 unique healthy skin samples were considered (Table 1).

In the first study, Lowe et al. (SRAdb id: SRP273719) [37] aimed to uncover genes related to HS immunogenesis and their influence on patients’ responsiveness to anti-tumor necrosis factor alpha (TNF-α) therapy. This study was conducted through transcriptome sequencing of lesioned and non-lesioned skin samples before and after anti-TNF-α therapy. Herein, we focused our attention on the RNA-Seq from samples collected before anti-TNF-α therapy. In summary, 26 HS lesioned skin samples and 16 healthy skin controls were included in our analyses.

In the second study, Gudjonsson et al. (SRAdb id: SRP272659) [38] focused on outlining the major dysregulated cell types and inflammatory pathways in HS. In order to evaluate abnormal inflammatory pathways, they performed skin and whole blood RNA-Seq, and scRNA-Seq. In addition, they compared their findings with RNA-Seq data from 28 psoriasis patients and 32 atopic dermatitis patients. In summary, they found 4797 DEGs within the HS skin RNA-Seq compared to healthy controls, and 332 DEGs within HS patients’ whole blood. From this study, we incorporated into our analysis 32 skin RNA-Seq samples; 22 from HS lesioned skin and 10 from healthy skin controls.

In the third study, Witte-Händel et al. (SRAdb id: SRP168930) [39] investigated HS skin lesions’ cytokine milieu and found *IL-1β* to be highly expressed. First, they quantified the expression levels of approximately 30 mediators in HS lesioned skin and compared the results with the expression patterns from these same mediators in healthy skin control and in psoriasis lesioned skin. After finding *IL-1β* to be upregulated in HS, they compared the results of HS lesioned skin’s RNA-Seq with the transcriptome of various cell types isolated from healthy patients’ biopsies that were exposed to IL-1β and observed transcriptomic overlapping profiles. RT-qPCR and ELISA analyses were also conducted. Our analysis included samples from three HS patients’ lesioned skin biopsy and four healthy individuals skin biopsy not previously exposed with IL-1β.

### 3.2. Meta-Analisis

We performed a meta-analysis, which resulted in 6445 annotated genes, and 3628 mDEGs with an FDR < 0.05 and |log2(fold change)| > 1. From these mDEGs, 1699 were upregulated and 1929 were downregulated (Table S1). Briefly, upregulated mDEGs that play a role in the maintenance of skin inflammation and structure, namely *DEFB4A/B* [40,41], *TCN1* [9], *MMP1* [42], *S100A7A* [43], and *MMP3* [44] appeared at the top of the overall meta-analysis rank product list. Alongside these mDEGs, other immune-related genes such as *TNF-α*, *IFN-γ*, *IL-1β*, *IL-36*, *IL-17*, *MZB1*, *CD19*, *CD79A*, *CXCL13*, *RETN* [45], and *RARRES2* [46], and skin structure-related genes particularly *PI3* [47], *SERPINB3/4* [48], *SERPINA1* [49], *KRT6/16* [50], and *SPRR1/2/3* [51] were observed. Conversely, the highest positions on the overall rank product list for the down-regulated mDEGs included *THRSP*, *AWAT2*, *DCD*, *UGT3A2*, and *WIF1*, genes associated with energetic metabolism and skin’s barrier function [52,53,54,55,56]. Down-regulation of these mDEGs as well as other genes, especially *AQP2/4/5/6/7/8*, *FOXA1*, and *ADIPOQ*, may impair these pathways.

Pathway enrichment analysis of the mDEGs by reactome resulted in 76 statistically significant enriched pathways (Table S2). We then selected the 20 pathways with the lowest FDR values and summarized them in Table 2. Pathways such as keratinization (FDR = 0.0049, gene ratio = 54/1663), extracellular matrix (ECM) organization (FDR = 2.42 *×* 10^−^^14^, gene ratio 109/1663), neutrophil degranulation (FDR = 5.25 × 10^−^^9^, gene ratio = 132/1663), PD-1 signaling (FDR = 1.5532 *×* 10^−^^9^, gene ratio = 18/1663), interferon gamma signaling (FDR = 0.00003, gene ratio = 58/1663), signaling by interleukins (FDR = 2.3270 *×* 10^−^^6^, gene ratio = 118/1663), complement cascade (*p* value = 0.00003, gene ratio = 25/1663), antimicrobial peptides (*p* value = 3.476 *×* 10^−^^6^, gene ratio = 30/1663), cell surface interactions at the vascular wall (*p* value = 8.1106 *×* 10^−^^6^, gene ratio = 47/1663), and g-protein coupled receptor (GPCR) ligand binding (*p* value = 5.2463 *×* 10^−^^9^, gene ratio = 126/1663) appeared enriched.

In addition, a pathway enrichment analysis by PANTHER database was conducted. Summarily, pathways with the highest percentage of upregulated mDEGs enclosed inflammation mediated by chemokine and cytokine, integrin and interleukin signaling, T and B cell activation, angiogenesis, and apoptosis (Figure S1). Where, pathways containing the highest percentage of down-regulated mDEGs included wnt signaling, angiogenesis, G-protein signaling, gonadotropin-releasing hormone receptor, and cadherin signaling (Figure S2).

We then performed a set analysis to extract DEGs commonly found in all three selected studies (saDEGs), as well as in both analyses (cDEGs). These analyses can be located in the supplementary data.

### 3.3. RNA-Seq Variant Calling

We performed the variant calling in the HS lesioned samples and controls. The pipeline identified 323,827 unique variants across all samples. Among them, 190,845 variants were annotated as known SNPs and Indels.

After filtering, a total of 73,134 genetic variants present in 12,665 genes were found only in patients, and thus selected as candidate variants for HS pathogenesis. Up to 39,709 novel variants not present on dbSNP and Gnomad were found and marked by an asterisk at the end of the information regarding amino acids change and reference gene at the column “AAChange.refGene”. These results are summarized in Table S3. Briefly, 63,235 single nucleotide variants (SNVs) and 9899 insertion/deletion variants (Indels) were detected across the patients’ transcriptomes. They were distributed as follows: 39,577 in 3′-UTR, 3577 in 5′-UTR, 2325 in splicing sites, 25,577 exonic, 22 non-coding RNA splicing, and 2056 non-coding RNA exonic. Among the exonic variants present only in patients, 11,890 were synonymous, 12,266 were nonsynonymous, 35 were start-loss, 140 were stop gain, 203 were non-frameshift deletions, 205 were non-frameshift insertions, 120 were frameshift deletions, and 651 were frameshift insertions.

The variant calling analysis identified 34 variants in genes of the γ-secretase complex. This complex, composed of 4 genes (presenilins (*PSEN*), nicastrin (*NCSTN*), anterior pharynx defective 1 (*APH1*), and presenilin enhancer 2 (*PSENEN*)), modulates NOTCH pathway by cleaving and releasing NOTCH’s intracellular domain [14], thus, being important for skin and immune response’s homeostasis and HS pathogenesis [57,58]. The 34 mutations were distributed as follows: 12 in the *NCSTN* gene, 3 in the *APH1A*, 6 in the *APH1B*, 8 in the *PSEN1*, 3 in the *PSEN2*, and 2 in the *PSENEN*. One variant was already reported in HS patients: a stop gain mutation NM_172341.4: c.168T>G (p.Tyr56X) of the *PSENEN* gene previously associated with concomitant HS and Dowling Degos Disease (DDD), a rare genodermatosis, classically characterized by acquired reticular hyperpigmentation in flexural sites [59]. Another non-synonymous variant NM_000243.2:c.2177T>C (p.Val726Ala) in the *MEFV* gene, previously associated with HS, has also been spotted [60]. In Table 3 we summarized these two mutations, along with other variants found in coding regions of genes already linked with HS [60,61,62].

Apart from variants in genes already considered related to the disease, a second table containing potential HS new genetic markers was created (Table 4). Overall, variants were observed in genes associated with cellular metabolism, immune response, and skin homeostasis. For instance, *ACSF3* plays a crucial role in fatty acid synthesis [63], and its dysregulation may affect the ability of the body to properly process certain proteins and lipids. In conjunction, we also observed mutations in genes, such as *BTN2A1*, *FNIP2*, *AKR1C3*, *ALDH6A1*, *YTHDF1*, and *GALNT7* that are similarly involved in the metabolism of lipids, proteins, fatty-acids, and sterols. Variants associated with immune response were spotted in genes such as *GSDMD*, *SIT1*, *WDR92*, and *GPANK1*. *GSDMD*, a gene that encodes protein Gasdermin D, is a positive regulator of *IL-1β* involved in pyroptosis cell death [64] and was found upregulated by our meta-analysis. SIT1 is involved in the regulation of T cell activation [65], while *WDR92* is involved in apoptosis via activation of caspase-3 [66]. Variants that impair apoptosis of keratinocytes may be associated with the hyperkeratosis responsible for hair follicle occlusion [67]. In the context of skin regulation, variants were detected in genes such as *KLF4*, *FLOT2*, *PHACTR4*, and *CORO1B*. *KLF4* is required for the normal development of the barrier function of skin. Moreover, KLF4 is also part of the NOTCH super pathway. *FLOT2* may act as a scaffolding protein and may be involved in epidermal adhesion, structure, and function, while *PHACTR4* and *CORO1B* behave as actin-binding proteins. Thus, variants in these genes could explain dysfunction of the skin barrier and structure. Finally, *MYOF* plays a role in the cell membrane repair mechanism of endothelial cells that permits rapid resealing of membranes disrupted by mechanical stress [68]. A decreased number of circulating progenitor endothelial cells and endothelial dysfunction in HS has been previously described [69,70].

The variants found by the variant calling corroborated abnormal pathways found by the meta-analysis. Therefore, loss or gain of function of these genes should be closely investigated to better understand their influence in the HS pathogenesis.

## 4. Discussion

**Figure 2:**
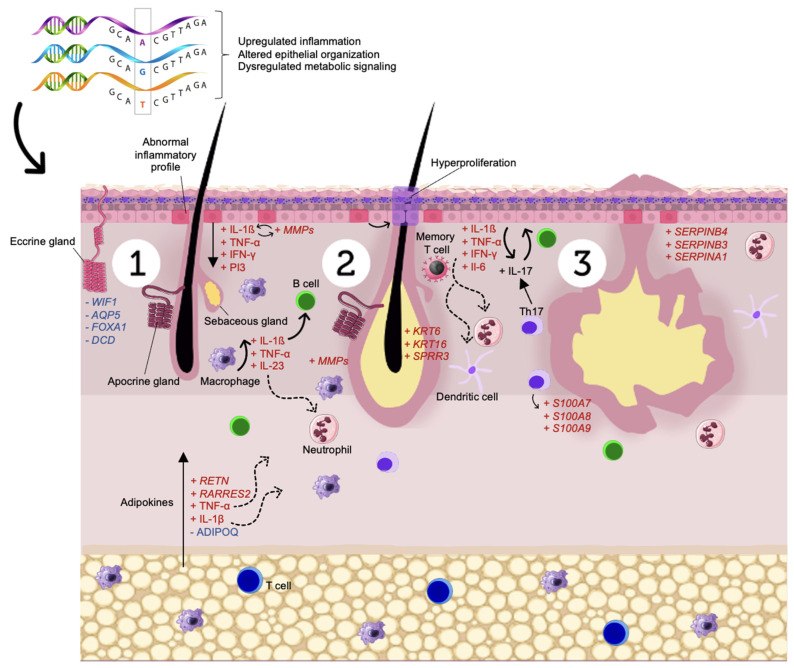
Representation of the HS pathogenesis based on the three main outcomes found by the meta-analysis: upregulated inflammation, altered epithelial organization, and dysregulated metabolic signaling; 1: It is believed that keratinocytes may have an abnormal inflammatory profile in HS lesions. These keratinocytes expressing cytokines such as TNF-α, IL-1β, IFN-γ, alongside pro-inflammatory adipokines, may induce infundibular hyperkeratosis and subsequent perifollicular immune cell infiltration; 2: Hyperkeratosis occludes the hair follicle, resulting in follicular hyperplasia and nodule formation demonstrated by upregulation of *KRT6-KRT16* and *SPRR3*; 3: Non-stop follicular dilatation leads to follicle rupture and impairment of the skin barrier function hinted at due to the upregulation of serpins-family genes, exacerbating inflammation. Recurrent injured nodules may evolve into dermal tunnels with impaired wound healing capacity, a consequence of abnormal eccrine gland function suggested by downregulation of *WIF1*, *AQP5*, *FOXA1*, and *DCD*.

Here, we operated an RNA-Seq meta-analysis to explore HS lesioned skin transcription profiles among three independent studies. These studies exploited HS immunopathogenesis, yet the re-evaluation of these transcriptomes resulted in more than immunological regulation, they highlighted other important DEGs in the context of skin homeostasis and energetic metabolism. The main findings are summarized in Figure 2, which briefly represents the three main outcomes of the meta-analysis at the molecular level: immune dysregulation, skin homeostasis perturbation, and abnormal metabolic signaling. In addition, we performed a variant calling from RNA-Seq data that may potentially contribute to novel insights for HS pathogenesis.

### 4.1. Immune Dysregulation

The importance of immunological pathways in HS is well accepted and their dysregulation may predispose the development of a strong initial inflammatory reaction commonly seen in HS patients [9]. Here, we provide evidence that many immune-associated DEGs are present in HS lesioned skin and absent in healthy control skin. These DEGs are crucial for commonly HS-associated enriched pathways, namely neutrophil degranulation, signaling by interleukins, complement cascade, immunoregulatory interactions between a lymphoid and a non-lymphoid cell, antimicrobial peptides, and interferon signaling.

Overall, our results were in agreement with the three studies used in our analyses. Lowe et al. [37], when comparing lesioned skin to healthy control, identified *IFN-γ* and *IL-1β* as highly upregulated DEGs. These genes were found to be more than 4 times overexpressed in the meta-analysis. Our results suggested that upregulation of both proinflammatory molecules was essential to local inflammation in the skin condition. In HS lesions, IFN-γ may function as the primary activator of macrophages, which were the most numerous immune cells in the inflammatory infiltrates [71]. In normal conditions, the recruitment of macrophages was crucial not only for the immune response but also for the removal of cellular debris, the promotion of healing, and reorganization of areas within inflammation [72]. However, dysregulated activation and proliferation of macrophages may contribute to an elevated secretion of pro-inflammatory cytokines, such as IL-1β [71]. Upregulation of *IL-1β* exacerbates the inflammation contributing to the recruitment of other immune cells to the lesion, and to the pus formation seen in HS abscess [73]. Indeed, interferon alpha/beta/gamma signaling, as well as neutrophil degranulation pathway appeared enriched by the reactome. Witte-Händel et al. [39], who also found *IL-1β* highly expressed in HS lesions, postulates that its pathways-related are of paramount importance for HS phenotype. Corroborating this theory, a potential gain-of-function mutation in a positive regulator of *IL-1β* (*GSDMD*), was found by the variant calling, and upregulated expression of important molecules for the IL-1 signaling, as well as genes that stimulate or are stimulated by the expression of this cytokine were found in the mDEGs.

Gudjonsson et al. [38] obtained similar results for *IFN-γ*, but not for *IL-1β*. Instead, they found upregulation of *IL36A* and *IL36G* genes. Still, these genes have been recently reported as agonistic cytokines in the interleukin IL-1 superfamily through the activation of nuclear factor-κB (NF-κB) and mitogen-activated protein kinase (MAPK) [74]. *IL-36A* and *IL-36G* were also found upregulated in the meta-analysis. Hessam S. et al. [74] provide evidence for a distinctive IL-36 pro-inflammatory role in the development of an inflammatory loop commonly seen in HS phenotypes. Besides, IL-36 was also found to induce HS like acanthosis and hyperkeratosis in transgenic mice [75].

In our analyses, we found the upregulation of *IL-17A* and *IL-17F*. In HS, the release of follicular debris in the dermis results in the activation of an immune response mediated by Th17 [76]. IL-17 induces proinflammatory cytokine expression by keratinocytes, contributing to immune cell infiltration by neutrophils, dendritic cells, and memory T cells to the lesioned area [77]. However, Kelly et al. proposed that Th17 cells were present in HS skin prior to the formation of an active lesion, indicating that they could participate in lesion development. Indeed, genes that are influenced by *IL-17*, such as *DEFB4A*/*B*, *MMP1*, and *MMP9* were also found upregulated, and had the highest-ranking product positions in the meta-analysis. Other skin genes, namely psoriasin (*S100A7*) and calprotectin (*S100A8/9*), whose expression augmented in the presence of IL-17, were also found upregulated in the meta-analysis. Since keratinocytes are believed to have an inflammatory profile in HS skin [78], these findings suggested that vicious interactions between keratinocytes and TH17/IL-17 contributed more to chronic inflammation than to enhancing a tissue protective response.

Enhanced B cell signatures were found by Lowe et al. [37] and Gudjonsson et al. [38] when they compared HS lesioned skin with the healthy control and other skin conditions, such as psoriasis. Although Witte-Händel et al. [39] did not explore B cell signaling, they mentioned finding upregulation of *CXCL13*, a B cell chemoattractant [79]. In our analyses, several genes associated with B cells, such as *MZB1*, *CD19*, CD79A, and CXCL13 were found to be expressed 10 times more in HS lesions when compared to controls. This may suggest that B cell alteration was a common event for HS development. B cells are important for HS pathogenesis since they contribute to fibrosis, stromal remodeling, and therapeutic responses [80]. The latter still remaining controversial; reports using rituximab, a monoclonal antibody that depletes B cells from the circulation targeting CD20 molecules, has shown to ameliorate HS phenotype [81] in some HS patients, but to induce HS-like lesions appearance in others [82]. When we analyzed the upregulated mDEGs in the PANTHER gene analysis tool, the B cell activation (P00010) pathway was enriched with 18 genes, suggesting an important role of B cells for HS lesions maintenance.

### 4.2. Skin Homeostasis

The main enriched pathways associated with skin regulation were ECM organization, keratinization, formation of the cornified envelope, epidermal cell differentiation, and collagen formation and degradation. Moreover, several variants associated with disruption of these pathways were identified, suggesting potential HS-related genes. Zouboulis C.C. et al. [9] demonstrated that the inflammatory process in HS was linked with abnormalities in these signaling routes. Our results confirmed these connections. For instance, elafin (*PI3*), an elastase-specific inhibitor that acted as an antimicrobial peptide and was expressed by epithelial and certain immune cells, appeared eight times more expressed. Besides acting against gram-positive and gram-negative bacteria, fungal pathogens, and being involved in NF-κB pathway modulation, cytokine secretion and cell recruitment [83], the gene encoding elafin has also been correlated to abnormal epithelial differentiation in a context of hyperproliferation [47,84]. Additionally, we found dysregulation in some genes of the serine protease inhibitor superfamily, serpin. Among them, *SERPINB4*, *SERPINB3*, and *SERPINA1* stood out in our ranking product, corroborating the involvement of the hair follicle unit [85]. Serpins overexpression has been previously spotted at the internal and epithelial root sheath of hair follicles in HS patients [9]. In general, serpins are associated with epidermal barrier homeostasis, and chronic skin inflammation [48]. These roles have been corroborated in atopic dermatitis and psoriasis [49,86], two inflammatory skin diseases that overlap molecular pathways with HS [87,88]. Thus, upregulation of *PI3* and *SERPINs* may be associated with keratinocyte hyperproliferation responsible for hair follicle plugin, a hallmark of HS.

Additionally, several cytokeratin appeared dysregulated, which may affect keratinization and formation of the cornified envelope. *KRT6* and *KRT16* are particularly interesting since they are expressed together in the outer root sheath of the hair follicles under stressful conditions [50]. In contrast, under homeostatic conditions, *KRT6-KRT16* are co-expressed to respond to barrier breach, stimulating hyperproliferation of interfollicular keratinocytes and modulating the inflammatory response to wounds [89]. Dysregulation of these KRTs have been previously assigned as psoriasis biomarkers [90,91]. In parallel, dysregulation of other mDEGs, such as *SPRR3*, and several subtypes of *SPRR2* and *SPRR1* also provided evidence of impairment of barrier functions in HS. SPRRs proteins play a structural role in the cornified envelope, and SPRR3 is generally not detected in normal skin [92]. It had been suggested that an altered expression of *SPRR3* could impact barrier function through altered production of cornified envelope scaffold that impaired the supramolecular organization of lamellar body-derived lipids into normal bilayer structures [93,94]. This type of dysregulation may be associated with thinning of the cornified envelope [95]. Thus, upregulation of these genes may be linked to the impaired structure and fragility of the cornified envelope and follicular infundibulum that easily break and start an immunological reaction.

Equally important to hair follicle fragility is the family of matrix metalloproteinases (MMPs), a zinc-dependent extracellular protease that break down and remodel the ECM [96]. MMPs play an important role in HS pathogenesis because it is involved in inflammation, sinus formation, and in the thinning of the basement membrane surrounding the hair follicle unit [97]. Interestingly, MMPs are also known to play a critical role in the conversion of IL-1β into its active form [98], which respond by enhancing MMP expression [99], potentially creating a long-lasting inflammatory response in HS lesioned skin. Here, we found up to 12 types of MMPs upregulated, of which *MMP1* and *MMP3* composed some of the highest positions in the meta-analysis overall ranking, suggesting that skin intrinsic components may represent a critical step in disease progression.

Finally, sweat glands are believed to regulate epidermal homeostasis and wound repair [100,101]. Genes that are relevant for a proper sweat-gland function namely *WIF1*, *AQP5*, *FOXA1*, and *DCD* were found highly decreased in HS skin by our meta-analysis. Respectively, the genes appeared approximately 10, 2, 6, and 24 times less expressed when compared to healthy skin. In normal conditions, sweat glands’ multipotent progenitor cells contribute to repair and skin re-epithelialization [100,101]. Thus, down-regulation of these genes may affect the capacity of skin repair, contributing to the clinical HS phenotype of non-healing wound-like environment. Similar results were found by Coates et al. (2019) [102], corroborating the possibility of impaired sweat gland function contributing to HS pathogenesis. Moreover, alongside *AQP5*, several other aquaporin-related genes such as *AQP7*, *AQP6*, *AQP8*, *AQP2*, *AQP4*, *AQP7P3*, *AQP7P1*, and *AQP4-AS1* were also found down-regulated in the mDEGs. Aquaporins are membrane channel proteins that through the bidirectional transport of water, glycerol, and small solutes across the membrane, serve as critical players of the skin barrier [103]. Besides their essential role for correct skin barrier activity, AQPs, mainly AQP7, also operated in the modulation of skin’s inflammatory responses [104]. In this context, the downregulation of several aquaporins may be associated with the impaired skin barrier function evidenced by our analyses and commonly seen in inflammatory skin diseases, as well as atypical skin immune surveillance.

### 4.3. Energy Metabolism

Metabolic dysregulation in HS was detected through several downregulated DEGs and pathways including the metabolism of lipids, triglyceride and fatty acid, and glucocorticoid biosynthesis. Additionally, variants found by the RNA variant calling may be associated with several metabolic-related disorders, including metabolic syndrome (MetS). MetS concomitant with HS had already been described [105,106,107]. Nevertheless, it is not clear if inflammation induced by MetS leads to the initiation of HS or if the systemic inflammation in HS leads to manifestations of MetS [106]. MetS is characterized by a combination of clinical conditions including central obesity, hyperglycemia, dyslipidemia, and/or hypertension [108,109]. Although not necessarily present, central obesity plays a critical role in MetS’ development [109], and phenotype severity in HS [110,111]. In fact, at the molecular level, metabolic disorders and obesity may be considered a primary risk factor in HS [112].

Body mass index (BMI) data was available for two studies. The data confirmed that most of the patients had a BMI > 25. Obesity is supposed to favor HS skin alteration in a few manners [113]. From the molecular point of view, obesity induces low levels of systemic inflammation and metabolic changes [97]. Inflammatory immune cells including M1-type macrophages and T cells infiltrate the hypertrophic and damaged adipose tissue, producing inflammatory cytokines and inducing a dysregulated pattern of soluble mediators called adipokines [114]. Adipokines seem to drive metabolic alterations at the same time it represents a mechanistic link in the interaction between skin and metabolic comorbidities [115]. They are regularly expressed by several skin cells, such as keratinocytes, melanocytes, sebocytes, and fibroblasts, and their abnormal expression has been associated with various inflammatory skin conditions [116]. A few types of adipokines have been described and can have good and bad properties regarding the skin and body’s homeostasis [116]. The “bad” adipokines include resistin (RETN), chemerin (RARRES2), and classical pro-inflammatory cytokines, such as TNF-α, and IL-1β [115]. These metabolic villains were found upregulated in our meta-analysis. Besides their role in skin inflammation with immune cell tissue infiltration and cell dysfunction, they also drive insulin resistance, disturbance of glucose and lipid metabolism, and vascular dysfunction [117,118,119,120]. The dysregulation of these pathways was corroborated by our findings, suggesting concomitant HS and metabolic-related disorders in the patients. On the other hand, “good” adipokines such as adiponectin (ADIPOQ) that have properties, such as control of fat metabolism and insulin sensitivity, with direct anti-diabetic, anti-atherogenic, and anti-inflammatory activities [117,121,122,123], were found ten times less expressed in HS lesions. This may represent an association between the downregulation of *ADIPOQ* and susceptibility of HS and metabolic comorbidities. The exploration of adipokines’ role in skin and cellular metabolism is still in an early phase.

## 5. Limitations

Not all the studies provided clinical information of the patients, such as weight, smoking history, and race, nor general information about lifestyle, comorbidities, and different HS phenotypes. Therefore, clinical information was not taken into consideration in our analysis. Still, independent studies provided similar results, confirming the importance of the DEGs found here for HS pathogenesis. Moreover, whole exome or genome sequencing data from the same samples were not available. Thus, we could not confirm the results found by our variant calling, nor could we confirm them by PCR or sanger sequencing. However, known HS-related variants were identified substantiating our findings. Ultimately, our study design involves re-analyzing previously published transcriptomic data and aggregating evidence of transcriptional changes, thus we were not able to confirm our results on the translational level. Notwithstanding, we hope our results and conclusions stimulate new functional studies that focus on confirming our findings at the protein/tissue level. In general, our results are in compliance with what is reported in the literature.

## 6. Conclusions

Herein, we confirm the HS pathogenic triad composed by upregulated inflammation, altered epithelial differentiation, and dysregulated metabolism signaling. The variant calling and meta-analysis verify this highly interconnected network. The upregulation of *PI3*, *TNF-α*, *IL-1β*, and *IFN-γ* suggest that the inflammatory process in HS may be linked with keratinocyte hyperproliferation. Moreover, the upregulation of *KRT6*, *KRT16*, serpin-family genes, and *SPRR3* alongside downregulation of aquaporin-family genes confirm the involvement of hair follicles and the impairment of barrier function in early phases of HS pathogenesis. Finally, our results demonstrate that HS and metabolic-related syndromes such as MetS and obesity may share transcription profiles and suggest that adipokines may be potential biomarkers for this interaction; down-regulation of *ADIPOQ* may be associated with diseases’ co-occurrence susceptibility. Several mutations associated with the HS pathogenic triad were found, highlighting potentially new HS-related genes associated with the sporadic form of this disease.

## Figures and Tables

**Table 1:** Detailed information regarding the three selected studies matching our study criteria.

SRA	Title	Samples Included in Our Study	Main Findings
SRP273719	Immunopathogenesis of hidradenitis suppurativa and response to anti–TNF-α therapy	42 samples (HS skin lesion pre-TNF = 19: HS skin lesion mild-moderate HS = 7: healthy skin control = 16)	Highly enriched pathways in HS lesioned skin are immune related. Signatures of complement activation, B cell signaling, and pathways involving phagocytosis were found to be unique to HS. TNF-α–regulated genes, *IFN-γ*, and *IL-1β* were selected as the major drivers of the inflammatory pathways in HS skin lesions. Nonetheless, IL-1 receptor antagonist, *IL-1RN*, and *IL-10RA*, 2 potent immunoregulatory molecules, were relatively reduced in HS skin. Alongside, α-catenin and sirtuin 1, both important for regulation of cell proliferation and survival, were reduced in HS skin.
SRP272659	Contribution of plasma cells and B cells to hidradenitis suppurativa pathogenesis	32 samples (HS skin lesion = 22: healthy skin control = 10)	Several upregulated genes in the skin were associated with B cell responses, including immunoglobulin genes such as *IGLV3-27*, *CD19*, and *CD79a*. Other important genes found were the antimicrobial gene *DEFB4A*; *CXCL13*, a B cell chemoattractant, and the neutrophil chemokine *CXCL1*. In summary, they found B cells, and in particular plasma cells, as a potential therapeutic target in HS.
SRP168930	The IL-1 pathway is hyperactive in hidradenitis suppurativa and contributes to skin infiltration and destruction	7 samples (HS skin lesion = 3: healthy skin control = 4)	*IL-1β* is highly active in HS, contributing to local and systemic inflammation. *IL-1β* induces expression of many molecules involved in extracellular matrix destruction including MMPs, ADAM12, serpinA1, COL3A1, and COL10A1, and immune cell infiltration such as CXCL1, CXCL6, CCL7, CXCL10, CXCL16, CXCL13, CCL24, CCL2, CCL8, and CCL20. *IL-1β* and, therefore, *MMP1*, *MMP3*, *MMP9*, *MMP10*, *CCL2*, *CXCL1*, *IL-6*, *and IL-32* were upregulated when compared with healthy control skin and psoriasis lesions.

**Table 2:** Reactome analysis highlighting the 20 pathways with the lowest FDR, which are associated with upregulated mDEGs.

Pathway Identifier	Pathway Name	Entities Found	FDR
R-HSA-198933	Immunoregulatory interactions between a lymphoid and a non-lymphoid cell	81	7.2839 × 10^−31^
R-HSA-1474244	Extracellular matrix organization	109	2.4259 × 10^−14^
R-HSA-380108	Chemokine receptors bind chemokines	35	1.4862 × 10^−11^
R-HSA-373076	Class A/1 (Rhodopsin-like receptors)	106	2.2223 × 10^−11^
R-HSA-202430	Translocation of ZAP-70 to immunological synapse	16	1.2195 × 10^−9^
R-HSA-389948	PD-1 signaling	18	1.55 × 10^−9^
R-HSA-6798695	Neutrophil degranulation	132	5.2463 × 10^−9^
R-HSA-500792	GPCR ligand binding	126	5.25 × 10^−9^
R-HSA-909733	Interferon alpha/beta signaling	33	5.2463 × 10^−9^
R-HSA-202433	Generation of second messenger molecules	22	5.2463 × 10^−9^
R-HSA-202427	Phosphorylation of CD3 and TCR zeta chains	17	5.2463 × 10^−9^
R-HSA-1474228	Degradation of the extracellular matrix	55	1.2723 × 10^−8^
R-HSA-877300	Interferon gamma signaling	40	3.5328 × 0^−8^
R-HSA-1442490	Collagen degradation	32	6.6811 × 10^−8^
R-HSA-375276	Peptide ligand-binding receptors	67	7.395 × 10^−8^
R-HSA-6809371	Formation of the cornified envelope	50	8.368 × 10^−8^
R-HSA-6785807	Interleukin-4 and Interleukin-13 signaling	44	1.1813 × 10^−7^
R-HSA-6783783	Interleukin-10 signaling	25	2.22 × 10^−7^
R-HSA-1474290	Collagen formation	37	7.0322 × 10^−7^
R-HSA-418594	G alpha (i) signaling events	108	1.4245 × 10^−6^

**Table 3:** Distribution of potential pathogenic variants with AF ≤ 0.01 among genes that have been already associated with HS.

Gene	DEGs	SNP ID	Ref	Alt	Distribution of Genotypes among HS Patients	Wilcoxon Test	Exonic Function	HGVS	AF
NCSTN	-	rs35603924	G	C	GG (50)/GC (1)	0.1087	nonsynonymous SNV	c.G231C:p.E77D	0.00432
APH1A	-	rs996158631	A	T	AA (50)/AT (1)	0.5630	nonsynonymous SNV	c.T123A:p.D41E	0.00010
APH1B	-	rs142676640	C	T	CC (50)/CT (1)	0.2229	nonsynonymous SNV	c.C640T:p.R214	0.000699
PSEN2	-	rs143912759	C	A	CC (50)/CA (1)	0.4288	nonsynonymous SNV	c.C1139A:p.T380K	0.00026
PSEN1	-	rs1174374799	-	T	−(50)/−T (1)	0.4542	frameshift insertion	c.526dupT:p.S178Ffs * 10	0.000006573
PSENEN	-	rs751542345	T	G	TT (50)/TG (1)	0.1519	stopgain	c.T168G:p.Y56X	0.000008
FGFR2	-	rs56226109	G	A	GG (49)/GA (2)	0.2413	nonsynonymous SNV	c.C170T:p.S57L	0.003722
MEFV	up	rs28940579	A	G	AA (50)/AG (1)	0.0215	nonsynonymous SNV	c.T2177C:p.V726A	0.001440
MEFV	up	rs104895094	T	C	TT (50)/TC (1)	0.0996	nonsynonymous SNV	c.A2084G:p.K695R	0.005245
NOD2	-	rs104895452	C	A	CC (50)/CA (1)	0.9148	nonsynonymous SNV	c.C2672A:p.A891D	0.000707
NOD2	-	rs5743279	G	A	GG (50)/GA (1)	0.1775	nonsynonymous SNV	c.G2288A:p.R763Q	0.001217
NOD2	-	rs5743272	A	G	AA (49)/AG (2)	0.0699	nonsynonymous SNV	c.A974G:p.H325R	0.000392
NOD2	-	rs35285618	G	A	GG (50)/GA (1)	0.2845	nonsynonymous SNV	c.G2042A:p.R681H	0.00198
NOD2	-	rs2066847	-	C	−(50)/−C (1)	0.6377	frameshift insertion	c.2936dupC:p.L980Pfs * 2	0.015002
NOD2	-	rs34684955	G	A	GG (50)/GA (1)	0.2845	nonsynonymous SNV	c.G337A:p.A113T	0.00251
NOD2	-	rs5743278	C	G	CC (50)/CG (1)	0.1038	nonsynonymous SNV	c.C2093G:p.A698G	0.00371
NOD2	-	rs576658764	C	T	CC (50)/CT (1)	0.2845	nonsynonymous SNV	c.C1540T:p.R514W	0.00007
PSTPIP1	up	rs34240327	G	C	GG (49)/GC (2)	0.4935	nonsynonymous SNV	c.G773C:p.G258A	0.00461

Ref = reference; Alt = altered; HGVS = Human Genome Variation Society; AF = allelic frequency.

**Table 4:** Potential Clin Var pathogenic non-synonymous variants found by the RNA-Seq variant calling analysis in HS patients with an allele frequency ≤ 0.01, CADD score > 15, and genotypic differences that were statistically significant (*p* value < 0.05).

Genes	DEGs	SNP ID	Ref	Alt	Distribution of Genotypes among HS Patients	Wilcoxon Test	HGVS	AF	CADD Score
ACSF3	-	rs144681140	G	A	GG (49)/GA (2)	0.0295	c.G1406A:p.R469Q	0.0032	22.9
KLF4	-	rs139237114	G	A	GG (49)/GA (2)	0.0385	c.C859T:p.H287Y	0.0016	23.8
DUSP23	-	rs11544443	A	T	AA (49)/AT (2)	0.0475	c.A371T:p.E124V	0.0022	25.1
BTN2A1	-	rs143104579	G	A	GG (48)/GA (3)	0.045	c.G188A:p.R63H	0.0096	18.01
FLOT2	-	rs3736238	C	T	CC (49)/CT (2)	0.0284	c.G982A:p.A328T	0.0119	18.44
GPANK1	-	rs35265780	G	A	GG (49)/GA (2)	0.04	c.C335T:p.A112V	0.0096	32
FNIP2	-	rs62001914	C	A	CC (49)/CA (2)	0.0476	c.C1653A:p.S551R	0.0092	24.8
CORO1B	-	rs145707942	C	G	CC (49)/CG (2)	0.0462	c.G367C:p.E123Q	0.0002	24.2
ADCY4	-	rs61745073	T	A	TT (49)/TA (2)	0.0357	c.A1358T:p.E453V	0.0022	22.7
AKR1C3	down	rs34186955	C	T	CC (49)/CT (2)	0.0295	c.C538T:p.P180S	0.0086	23.3
ALDH6A1	down	rs139579994	G	A	GG (49)/GA (2)	0.0395	c.C716T:p.P239L	0.0018	27.9
GSDMD	up	rs62000416	C	A	CC (49)/CA (2)	0.0315	c.C556A:p.L186M	0.0056	23.4
YTHDF1	-	rs141487890	G	A	GG (49)/GA (2)	0.0344	c.C437T:p.A146V	0.0008	24
MYOF	-	rs61861290	G	A	GG (48)/GA (3)	0.0207	c.C4576T:p.P1526S	0.0062	26.2
SIT1	up	rs138786883	C	A	CC (49)/CA (2)	0.0496	c.G520T:p.A174S	0.0032	17.54
RBMXL1	-	rs139713926	T	C	TT (49)/TC (2)	0.0242	c.A701G:p.Y234C	0.0022	25.5
GALNT7	-	rs144873913	C	A	CC (48)/CA (2)	0.0496	c.C1585A:p.P529T	0.0014	30
PHACTR4	-	rs72661785	G	C	GG (49)/GC (2)	0.043	c.G1609C:p.A537P	0.0036	26.3
WDR92	-	rs138784630	C	T	CC (49)CT (2)	0.04	c.G841A:p.A281T	0.0072	23.4

Ref = reference; Alt = altered; HGVS = Human Genome Variation Society sequence variant nomenclature; AF = allelic frequency; CADD score = mutation impact prediction.

## Data Availability

Transcriptomic data sets can be accessed at the Sequence Read Archive (SRA) database and Gene Expression Omnibus (GEO) database (SRP273719/GSE155176, SRP272659/GSE154773, and SRP168930/GSE122592), both from the National Center for Biotechnology Information.

## Supplementary Materials

The following supporting information can be downloaded at: https://www.mdpi.com/article/10.3390/biom12101371/s1, Figure S1: Percent of upregulated gene hits found in the mDEGs against total pathway hits, *p*-value < 0.05; Figure S2: Percent of down-regulated gene hits found in the mDEGs against total pathway hits, *p*-value < 0.05; Figure S3: Venn Diagram analysis representing saDEGs found in the individual studies; (a) saDEGs of the included studies; (b) upregulated saDEGs; (c) downregulated saDEGs. Figure S4: Percent of upregulated gene hits against total pathway hits; Table S1: Total mDEGs; Table S2: Reactome enriched pathways analysis associated with mDEGs (FDR < 0.05); Table S3: Variants found in patients only by the RNA-Seq variant calling; Table S4: Enriched pathways associated with saDEGs, and therefore, cDEGs, filtered by FDR < 0.05 found in the set analysis. Among the upregulated cDEGs, 91 genes had major roles in immunological pathways. For instance, 34 genes, such as *CYBB* (*p*-value = 1.758 × 10^−20^, logfc = 3.1303), *CD3E* (*p* value = 2.421 × 10^−15^, logfc = 2.6583), *CD8A* (*p* value = 3.734, logfc = 2.2508), and *CD19* (*p* value = 2.485 × 10^−30^, logfc = 5.5543) were associated with adaptive immune response and their cellular signaling networks, while 44 genes, including Matrix Metallopeptidases (MMPs) family genes, *DEFB4* (*p* value = 2.278 × 10^−58^, logfc = 7.6490), *TLR8* (*p* value = 3.003 × 10^−19^, logfc = 2.8510), and *BIN2* (*p* value = 2.563 × 10^−13^, logfc = 2.4414), were linked with innate immune system. In other important pathways, such as extracellular matrix organization, 23 genes such as *ADAM12* (*p* value = 1.492 × 10^−22^, logfc = 4.1200), *ADAMTS2* (*p* value = 2.6091, logfc = 1.111 × 10^−14^), and *COL3A1* (*p* value = 1.433 × 10^−17^, logfc = 2.7178), were found. On the other hand, down-regulated cDEGs, such as *FA2H* (*p* value = 5.394 × 10^−20^, logfc = −3.7949), *FADS2* (*p* value =1.073 × 10^−24^, logfc = −4.61), *GUCY2EP* (*p* value = 5.673, logfc = −1.9362), *HSD3B1* (*p* value = 4.861 × 10^−30^, logfc = −5.2634), *IYD* (*p* value = 1.337 × 10^−20^, logfc = −2.4972), *MOGAT2* (*p* value = 1.59 × 10^−23^, logfc = −3.7538), *MPPED1* (*p* value = 1.519 × 10^−25^, logfc = −3.1882), *PNPLA5* (*p* value = 3.314 × 10^−29^, logfc = −4.2126), *SOX9-AS1* (*p* value = 8.702 × 10^−17^, logfc = −2.3383), and *THRSP* (*p* value = 4.101 × 10^−48^, logfc = −5.4498) play a crucial role in metabolic pathways. In particular, *FA2H*, *FADS2*, *MOGAT2*, *HSD3B1*, *THRSP*, and *PNPLA5* are important for the metabolism of lipids while *IYD* participates in thyroxine biosynthesis and metabolism of amine-derived hormones.
