# Supplementary material for: Transcriptome Meta-Analysis Confirms the Hidradenitis Suppurativa Pathogenic Triad: Upregulated Inflammation, Altered Epithelial Organization, and Dysregulated Metabolic Signaling
Source: Biomolecules. 2022 Sep 25;12(10):1371. doi: 10.3390/biom12101371 (PMC9599370; doi:10.3390/biom12101371)
Supplement: Supplementary file 1 [file biomolecules-12-01371-s001.zip › Supplementary_Data.pdf]

Supplementary Data

Although the Set Analysis is a less powerful analysis than the meta-analysis, we conducted it to evaluate common genes between all independent studies. DEGs found in this analysis could be constantly associated with HS lesion formation, and it may provide important insights for a better understanding of HS pathogenesis. Moreover, since the meta-analysis resulted in over 3,000 mDEGs, a shorter number provided by saDEGs and cDEGs could help in easing the understanding of this complex disease.

Materials and Methods

Set Analysis

The lists of DEGs from each study resulting from the previous steps were compared by Set Analysis. Using structured query language (SQL), we created a cross-reference DEGs table containing all the common genes found in the set analysis (saDEGs) of the studies. The Set Analysis, and their corresponding Venn diagrams were generated with the VennDiagram package for R software version 4.1.0.

Pathway Analysis

We performed a pathway analysis with the saDEGs. We searched the REACTOME database, using ReactomePA package [24] for R software, and PANTHER v17.0 [29,30]. Results with FDR-adjusted enrichment test *p*-value < 0.05 were considered significant.

Results

The Set Analysis resulted in 232 saDEGs prevailing in HS lesions (Table S1 and Figure S3), of which 219 were upregulated and 13 were downregulated. To note, all these genes were also observed in the mDEGs. Therefore, these saDEGs resulting from the intersection of all studies were retrieved in both analyses (cDEGs). Moreover, the reactome pathway analysis of these 232 cDEGs revealed 37 enriched pathways with an FDR value < 0.05, of which 20 were upregulated and 16, downregulated (Table S2). Overall, the results of upregulated pathways were congruent with what was seen in the meta-analysis findings. In fact, 16 of the pathways found by the reactome in the Set Analysis were also noticed in the meta-analysis. Some of the enriched upregulated pathways found were immune system, ECM organization and degradation, peptide ligand-binding receptors, collagen formation and degradation, and class A/1 (Rhodopsin-like receptors). On the other hand, pathways related to general cellular metabolism were downregulated. We then analyzed the upregulated cDEGs with the PANTHER pathway database. The results are summarized in Figure S4. Pathways associated with B cell activation, integrin signaling, inflammation mediated by chemokine and cytokine signaling, and integrin signaling were the pathways hit with the highest percentage of genes. No hits of the down-regulated cDEGs were found by the PANTHER Pathway tool.

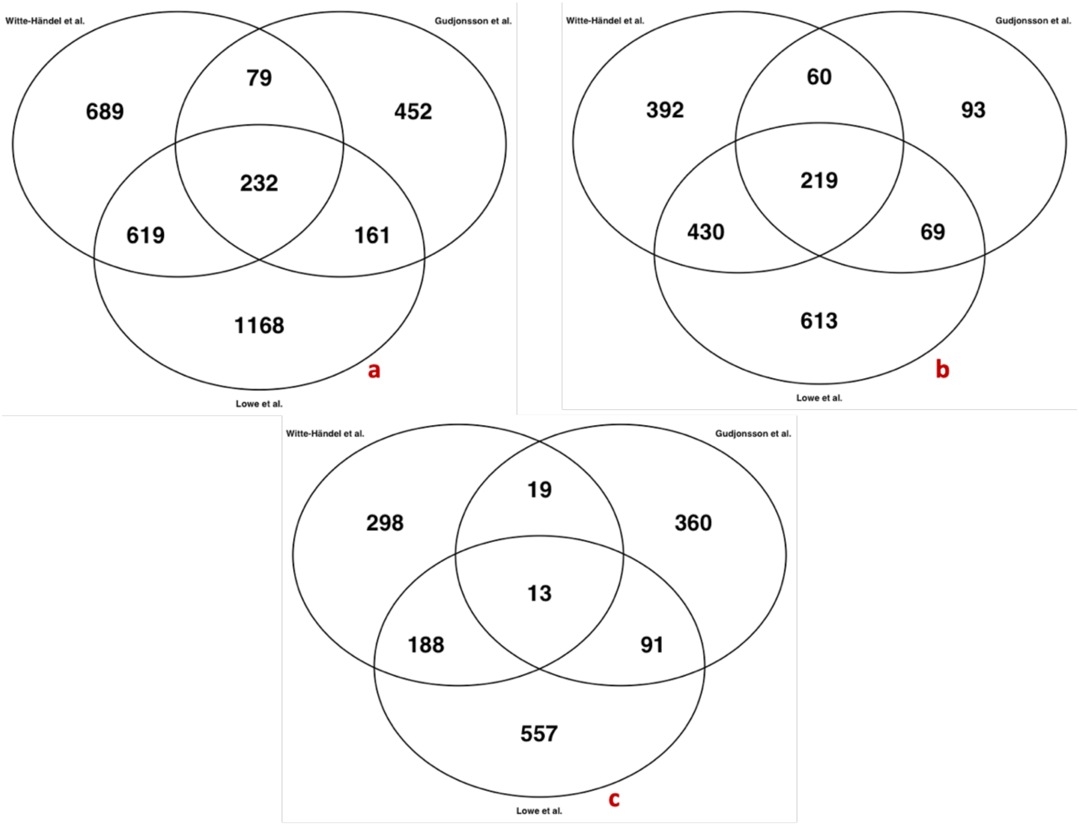

Figure S3. Venn Diagram analysis representing saDEGs found in the individual studies. a saDEGs of the included studies; b upregulated saDEGs. c downregulated saDEGs.

Table S4. Enriched pathways associated with saDEGs, and therefore cDEGs, filtered by FDR < 0.05 found in the set analysis. Among the upregulated cDEGs, 91 genes had major roles in immunological pathways. For instance, 34 genes such as *CYBB* (p-value = 1.758 x 10^{-20}, logfc = 3.1303), *CD3E* (p value = 2.421 x 10^{-15}, logfc =2.6583) , *CD8A* (p value = 3.734, logfc = 2.2508), *CD19* (p value = 2.485 x10^{-30}, logfc = 5.5543) were associated with adaptive immune response and their cellular signaling networks, while 44 genes, including Matrix Metallopeptidases (MMPs) family genes, *DEFB4* (p value = 2.278 x 10^{-58}, logfc = 7.6490), *TLR8* (p value = 3,003 x 10^{-19}, logfc = 2.8510), *BIN2* (p value = 2.563 x 10^{-13}, logfc = 2.4414), were linked with innate immune system. In other important pathways such as extracellular matrix organization, 23 genes like *ADAM12* (p value = 1.492 x 10^{-22}, logfc = 4.1200), *ADAMTS2* (p value = 2.6091, logfc = 1.111 x 10^{-14}) and *COL3A1* (p value = 1.433 x 10^{-17}, logfc = 2.7178), were found. On the other hand, down-regulated cDEGs such as *FA2H* (p value = 5.394 x 10^{-20}, logfc = -3.7949), *FADS2* (p value =1.073 x 10^{-24}, logfc = -4.61), *GUCY2EP* (p value = 5.673, logfc = -1.9362), *HSD3B1* (p value = 4.861 x10^{-30}, logfc = -5.2634), *IYD* (p value = 1.337x 10^{-20}, logfc = -2.4972), *MOGAT2* (p value = 1.59 x10^{-23}, logfc = -3.7538), *MPPED1* (p value = 1.519 x10^{-25}, logfc = -3.1882), *PNPLA5* (p value = 3.314 x10^{-29}, logfc = -4.2126), *SOX9-AS1* (p value = 8.702 x 10^{-17}, logfc = -2,3383), *THRSP* (p value = 4.101 x 10-48, logfc = -5.4498), play a crucial role in metabolic pathways. In particular, *FA2H*, *FADS2*, *MOGAT2*, *HSD3B1*, *THRSP*, *PNPLA5* are important for the metabolism of lipids while *IYD* participates in thyroxine biosynthesis and metabolism of amine-derived hormones.

Pathway identifier	Pathway name	Pathway also found in by the meta-analysis	Entities found	saDEGs	FDR
R-HSA-168256	Immune System	No	91	Up	7.37x10^{-09}

R-HSA-380108	Chemokine receptors bind chemokines	Yes	13	Up	$7.37x10^{-09}$
R-HSA-198933	Immunoregulatory interactions between a Lymphoid and a non-Lymphoid cell	Yes	25	Up	$5.48x10^{-08}$
R-HSA-1474244	Extracellular matrix organization	Yes	23	Up	$2.01x10^{-06}$
R-HSA-8949275	RUNX3 Regulates Immune Response and Cell Migration	No	6	Up	$2.12x10^{-06}$
R-HSA-6783783	Interleukin-10 signaling	Yes	11	Up	$2.57x10^{-05}$
R-HSA-375276	Peptide ligand-binding receptors	Yes	16	Up	$3.60x10^{-05}$
R-HSA-1474228	Degradation of the extracellular matrix	Yes	13	Up	$1.10x10^{-04}$
R-HSA-1442490	Collagen degradation	Yes	9	Up	$1.64x10^{-04}$
R-HSA-449147	Signaling by Interleukins	Yes	28	Up	$3.11x10^{-04}$
R-HSA-1474290	Collagen formation	Yes	10	Up	$5.49x10^{-04}$
R-HSA-2022090	Assembly of collagen fibrils and other multimeric structures	Yes	8	Up	$7.33x10^{-04}$
R-HSA-168249	Innate Immune System	No	44	Up	$7.33x10^{-04}$
R-HSA-1592389	Activation of Matrix Metalloproteinases	Yes	6	Up	$9.57x10^{-04}$
R-HSA-6798695	Neutrophil degranulation	Yes	21	Up	0.00260791
R-HSA-373076	Class A/1 (Rhodopsin-like receptors)	Yes	19	Up	0.00272173
R-HSA-216083	Integrin cell surface interactions	Yes	8	Up	0.00306491
R-HSA-1280218	Adaptive Immune System	No	34	Up	0.00306491
R-HSA-8939245	RUNX1 regulates transcription of genes involved in BCR signaling	No	3	Up	0.00487645
R-HSA-1280215	Cytokine Signaling in Immune system	No	34	Up	0.01218292
R-HSA-556833	Metabolism of lipids	No	6	Down	0.00202704
R-HSA-8979227	Triglyceride metabolism	No	2	Down	0.0087015
R-HSA-1430728	Metabolism	No	7	Down	0.01607597
R-HSA-193993	Mineralocorticoid biosynthesis	No	1	Down	0.01607597
R-HSA-2046105	Linoleic acid (LA) metabolism	No	1	Down	0.01607597

R-HSA-194002	Glucocorticoid biosynthesis	No	1	Down	0.01607597
R-HSA-209968	Thyroxine biosynthesis	No	1	Down	0.01607597
R-HSA-193048	Androgen biosynthesis	No	1	Down	0.01607597
R-HSA-2046104	alpha-linolenic (omega3) and linoleic (omega6) acid metabolism	No	1	Down	0.01607597
R-HSA-2046106	alpha-linolenic acid (ALA) metabolism	No	1	Down	0.01607597
R-HSA-200425	Carnitine metabolism	No	1	Down	0.01607597
R-HSA-75109	Triglyceride biosynthesis	No	1	Down	0.01607597
R-HSA-8978868	Fatty acid metabolism	Yes	2	Down	0.01761335
R-HSA-209776	Metabolism of amine-derived hormones	Yes	1	Down	0.02062509
R-HSA-163560	Triglyceride catabolism	No	1	Down	0.02741238
R-HSA-196071	Metabolism of steroid hormones	No	1	Down	0.0397431

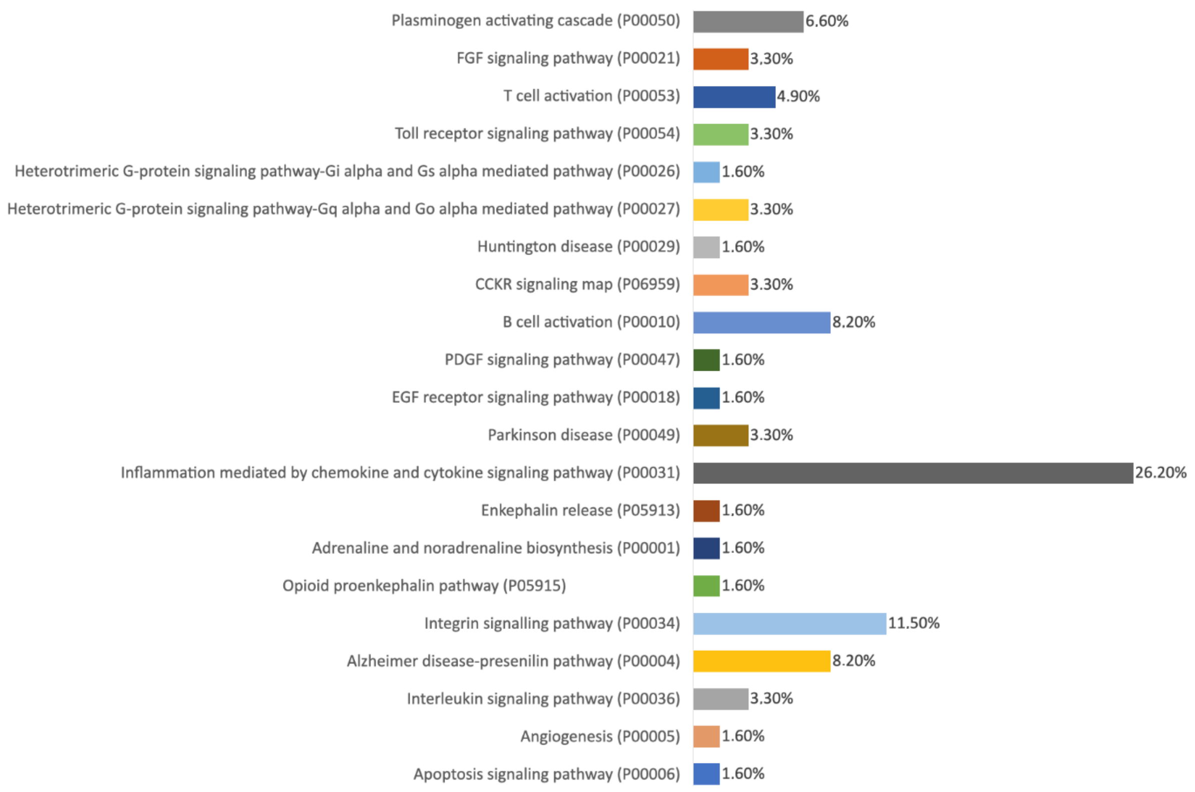

Figure S4. Percent of upregulated gene hits against total pathway hits.
