# Supplementary material for: Transcriptome Meta-Analysis Confirms the Hidradenitis Suppurativa Pathogenic Triad: Upregulated Inflammation, Altered Epithelial Organization, and Dysregulated Metabolic Signaling
Source: Biomolecules. 2022 Sep 25;12(10):1371. doi: 10.3390/biom12101371 (PMC9599370; doi:10.3390/biom12101371)
Supplement: Supplementary file 1 [file biomolecules-12-01371-s001.zip › TableS4-MDPI.pdf]

**Table S4.** Enriched pathways associated with saDEGs, and therefore cDEGs, filtered by FDR < 0.05 found in the set analysis. Among the upregulated cDEGs, 91 genes had major roles in immunological pathways. For instance, 34 genes such as *CYBB* (p-value = 1.758 x $10^{-20}$, logfc = 3.1303), *CD3E* (p value = 2.421 x $10^{-15}$, logfc =2.6583) , *CD8A* (p value = 3.734, logfc = 2.2508), *CD19* (p value = 2.485 x$10^{-30}$, logfc = 5.5543) were associated with adaptive immune response and their cellular signaling networks, while 44 genes, including Matrix Metallopeptidases (MMPs) family genes, *DEFB4* (p value = 2.278 x $10^{-58}$, logfc = 7.6490), *TLR8* (p value = 3,003 x $10^{-19}$, logfc = 2.8510), *BIN2* (p value = 2.563 x $10^{-13}$, logfc = 2.4414), were linked with innate immune system. In other important pathways such as extracellular matrix organization, 23 genes like *ADAM12* (p value = 1.492 x $10^{-22}$, logfc = 4.1200), *ADAMTS2* (p value = 2.6091, logfc = 1.111 x $10^{-14}$) and *COL3A1* (p value = 1.433 x $10^{-17}$, logfc = 2.7178), were found. On the other hand, down-regulated cDEGs such as *FA2H* (p value = 5.394 x $10^{-20}$, logfc = -3.7949), *FADS2* (p value =1.073 x $10^{-24}$, logfc = -4.61), *GUCY2EP* (p value = 5.673, logfc = -1.9362), *HSD3B1* (p value = 4.861 x$10^{-30}$, logfc = -5.2634), *IYD* (p value = 1.337x $10^{-20}$, logfc = -2.4972), *MOGAT2* (p value = 1.59 x$10^{-23}$, logfc = -3.7538), *MPPED1* (p value = 1.519 x$10^{-25}$, logfc = -3.1882), *PNPLA5* (p value = 3.314 x$10^{-29}$, logfc = -4.2126), *SOX9-AS1* (p value = 8.702 x $10^{-17}$, logfc = -2,3383), *THRSP* (p value = 4.101 x 10-48, logfc = -5.4498), play a crucial role in metabolic pathways. In particular, *FA2H, FADS2, MOGAT2, HSD3B1, THRSP, PNPLA5* are important for the metabolism of lipids while *IYD* participates in thyroxine biosynthesis and metabolism of amine-derived hormones.

| Pathway identifier | Pathway name | Pathway also found in the meta-analysis | Entities found | saDEGs | FDR |
|---|---|---|---|---|---|
| R-HSA-168256 | Immune System | No | 91 | Up | 7.37x$10^{-09}$ |
| R-HSA-380108 | Chemokine receptors bind chemokines | Yes | 13 | Up | 7.37x$10^{-09}$ |
| R-HSA-198933 | Immunoregulatory interactions between a Lymphoid and a non-Lymphoid cell | Yes | 25 | Up | 5.48x$10^{-08}$ |
| R-HSA-1474244 | Extracellular matrix organization | Yes | 23 | Up | 2.01x$10^{-06}$ |
| R-HSA-8949275 | RUNX3 Regulates Immune Response and Cell Migration | No | 6 | Up | 2.12x$10^{-06}$ |
| R-HSA-6783783 | Interleukin-10 signaling | Yes | 11 | Up | 2.57x$10^{-05}$ |

| | | | | | |
|---|---|---|---|---|---|
| R-HSA-375276 | Peptide ligand-binding receptors | Yes | 16 | Up | $3.60x10^{-05}$ |
| R-HSA-1474228 | Degradation of the extracellular matrix | Yes | 13 | Up | $1.10x10^{-04}$ |
| R-HSA-1442490 | Collagen degradation | Yes | 9 | Up | $1.64x10^{-04}$ |
| R-HSA-449147 | Signaling by Interleukins | Yes | 28 | Up | $3.11x10^{-04}$ |
| R-HSA-1474290 | Collagen formation | Yes | 10 | Up | $5.49x10^{-04}$ |
| R-HSA-2022090 | Assembly of collagen fibrils and other multimeric structures | Yes | 8 | Up | $7.33x10^{-04}$ |
| R-HSA-168249 | Innate Immune System | No | 44 | Up | $7.33x10^{-04}$ |
| R-HSA-1592389 | Activation of Matrix Metalloproteinases | Yes | 6 | Up | $9.57x10^{-04}$ |
| R-HSA-6798695 | Neutrophil degranulation | Yes | 21 | Up | 0.00260791 |
| R-HSA-373076 | Class A/1 (Rhodopsin-like receptors) | Yes | 19 | Up | 0.00272173 |
| R-HSA-216083 | Integrin cell surface interactions | Yes | 8 | Up | 0.00306491 |
| R-HSA-1280218 | Adaptive Immune System | No | 34 | Up | 0.00306491 |
| R-HSA-8939245 | RUNX1 regulates transcription of genes involved in BCR signaling | No | 3 | Up | 0.00487645 |
| R-HSA-1280215 | Cytokine Signaling in Immune system | No | 34 | Up | 0.01218292 |
| R-HSA-556833 | Metabolism of lipids | No | 6 | Down | 0.00202704 |
| R-HSA-8979227 | Triglyceride metabolism | No | 2 | Down | 0.0087015 |
| R-HSA-1430728 | Metabolism | No | 7 | Down | 0.01607597 |

| | | | | | |
|---|---|---|---|---|---|
| R-HSA-193993 | Mineralocorticoid biosynthesis | No | 1 | Down | 0.01607597 |
| R-HSA-2046105 | Linoleic acid (LA) metabolism | No | 1 | Down | 0.01607597 |
| R-HSA-194002 | Glucocorticoid biosynthesis | No | 1 | Down | 0.01607597 |
| R-HSA-209968 | Thyroxine biosynthesis | No | 1 | Down | 0.01607597 |
| R-HSA-193048 | Androgen biosynthesis | No | 1 | Down | 0.01607597 |
| R-HSA-2046104 | alpha-linolenic (omega3) and linoleic (omega6) acid metabolism | No | 1 | Down | 0.01607597 |
| R-HSA-2046106 | alpha-linolenic acid (ALA) metabolism | No | 1 | Down | 0.01607597 |
| R-HSA-200425 | Carnitine metabolism | No | 1 | Down | 0.01607597 |
| R-HSA-75109 | Triglyceride biosynthesis | No | 1 | Down | 0.01607597 |
| R-HSA-8978868 | Fatty acid metabolism | Yes | 2 | Down | 0.01761335 |
| R-HSA-209776 | Metabolism of amine-derived hormones | Yes | 1 | Down | 0.02062509 |
| R-HSA-163560 | Triglyceride catabolism | No | 1 | Down | 0.02741238 |
| R-HSA-196071 | Metabolism of steroid hormones | No | 1 | Down | 0.0397431 |
