# Supplementary figures and images for: Transcriptome Meta-Analysis Confirms the Hidradenitis Suppurativa Pathogenic Triad: Upregulated Inflammation, Altered Epithelial Organization, and Dysregulated Metabolic Signaling
Source: Biomolecules. 2022 Sep 25;12(10):1371. doi: 10.3390/biom12101371 (PMC9599370; doi:10.3390/biom12101371)

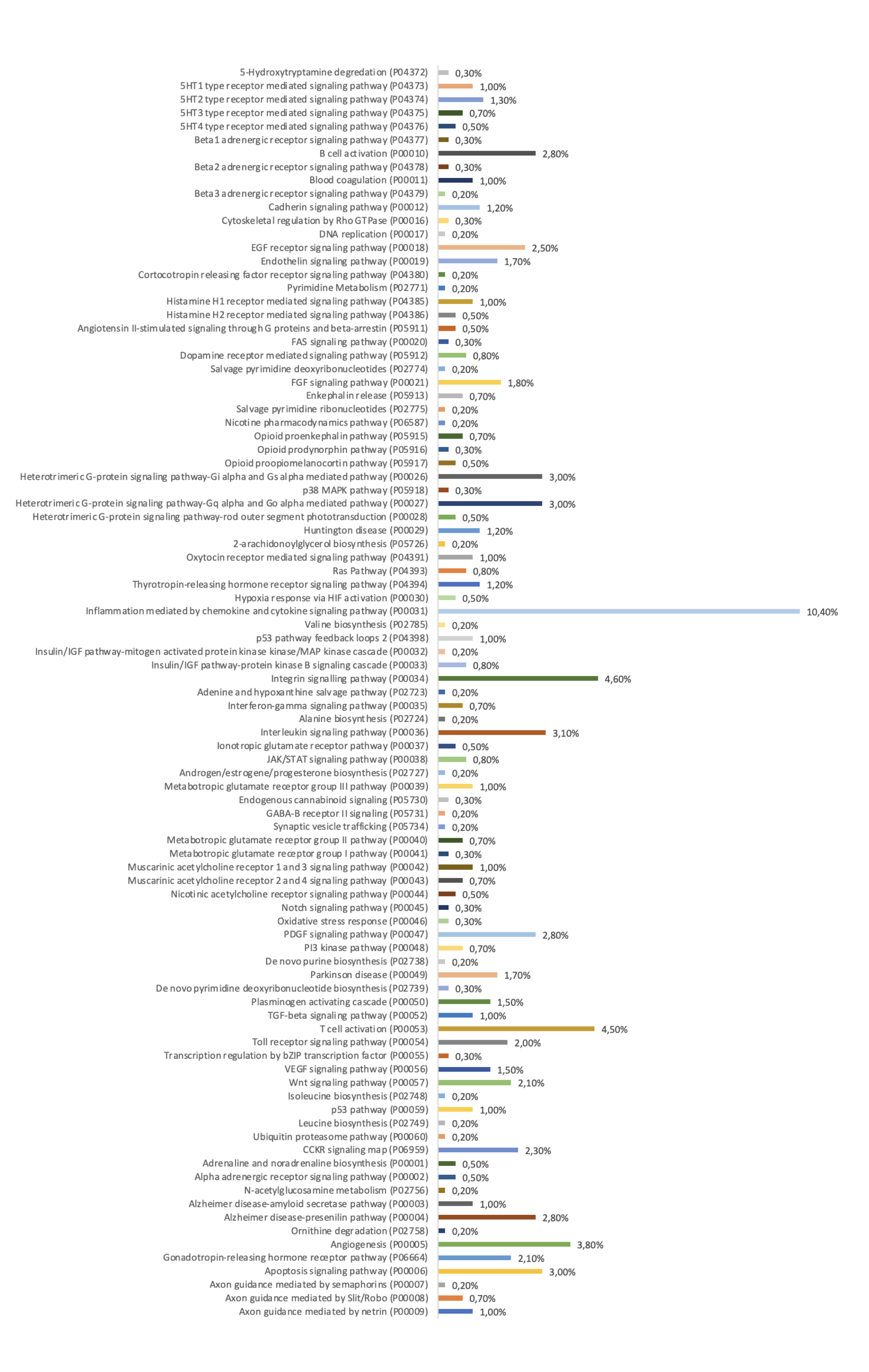

Supplement: Supplementary file 1 [file biomolecules-12-01371-s001.zip › FigS1-MDPI.tiff]

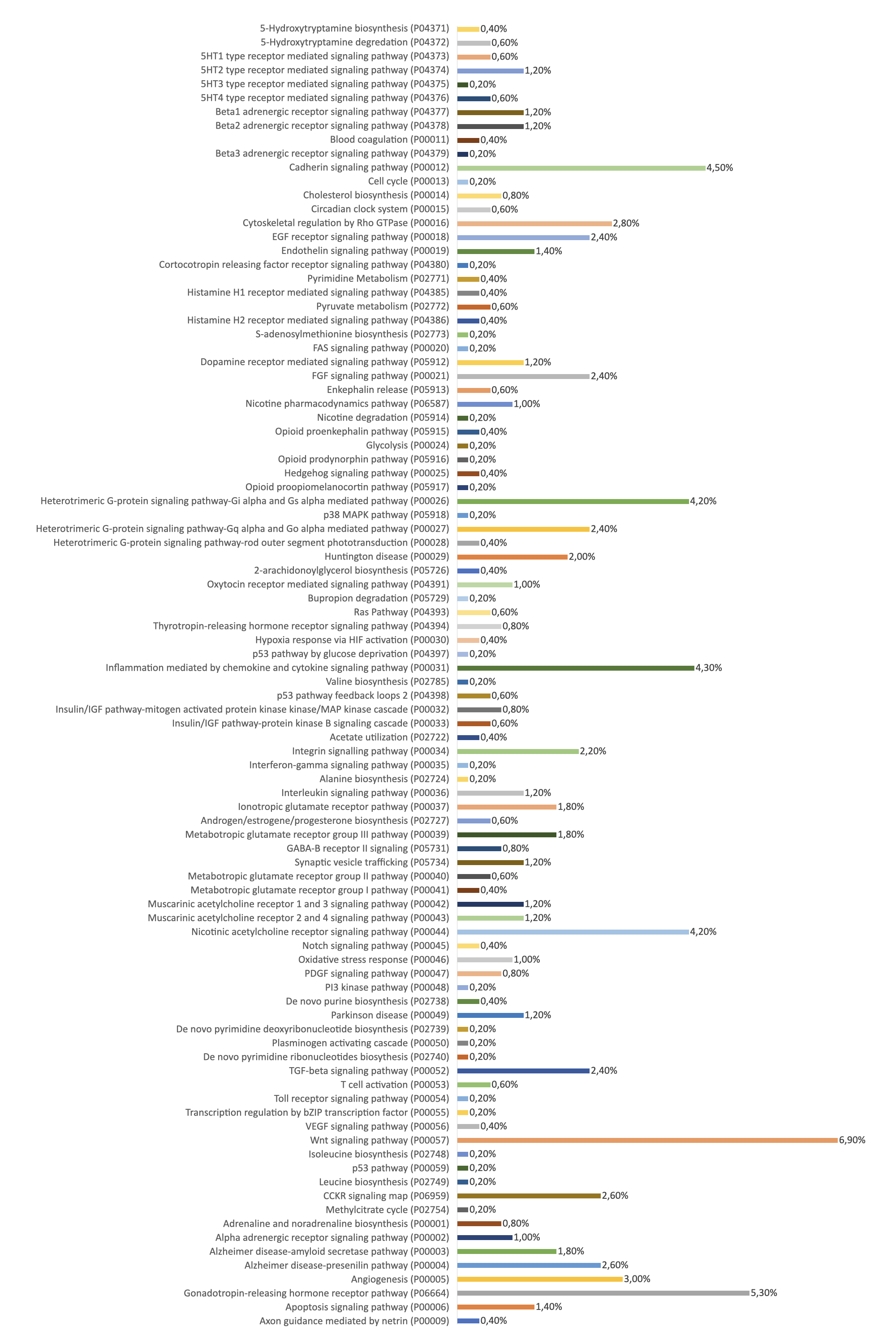

Supplement: Supplementary file 1 [file biomolecules-12-01371-s001.zip › FigS2-MDPI.tiff]

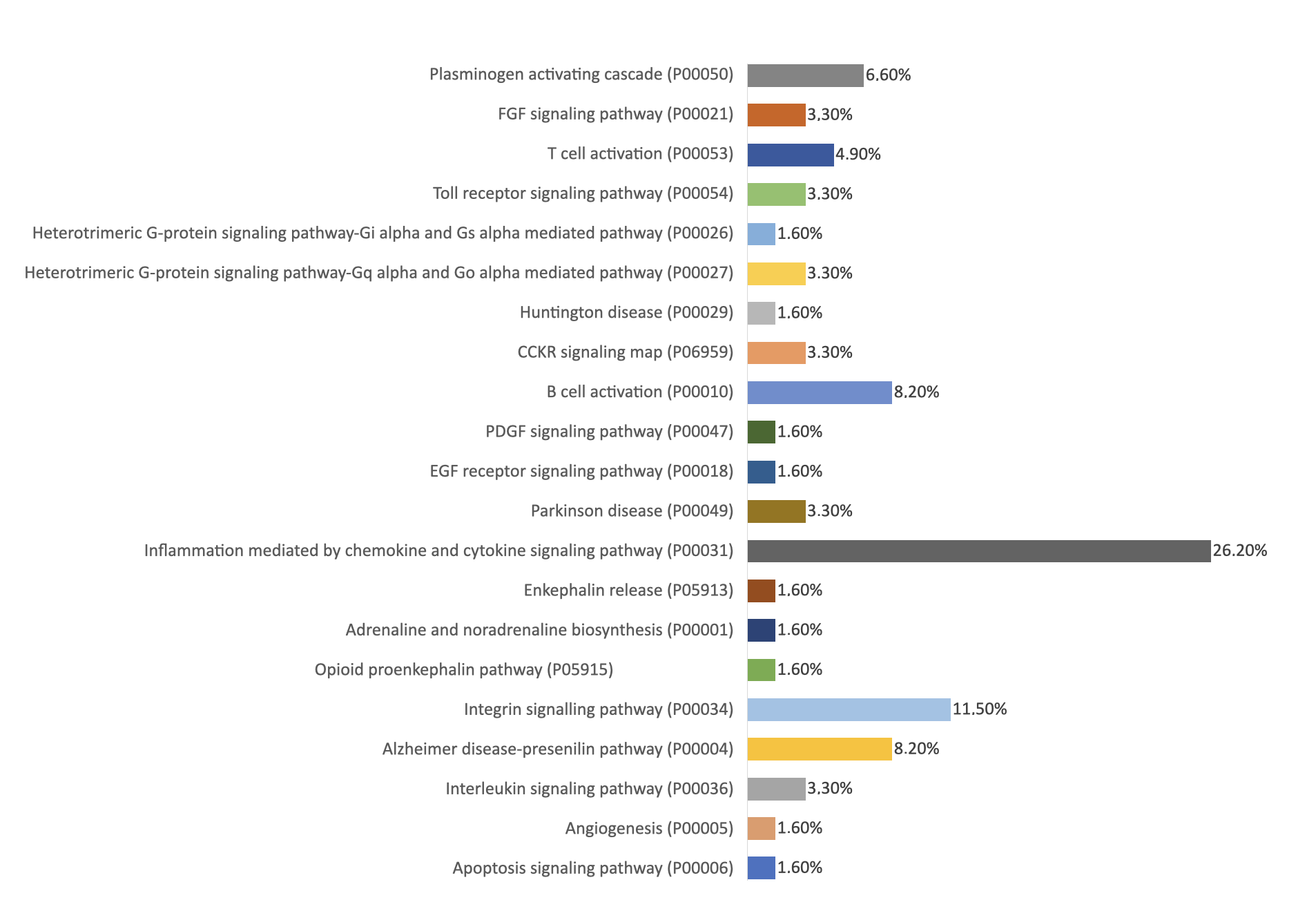

Supplement: Supplementary file 1 [file biomolecules-12-01371-s001.zip › FigS4-MDPI.tiff]
